# Correction: Understanding the quality of life of people living with HIV in rural and urban areas in Indonesia

**DOI:** 10.1371/journal.pone.0315033

**Published:** 2024-12-03

**Authors:** Nelsensius Klau Fauk, Hailay Abrha Gesesew, Lillian Mwanri, Karen Hawke, Paul Russell Ward

There is an error in affiliation 1 for authors Nelsensius Klau Fauk, Hailay Abrha Gesesew, Lillian Mwanri and Paul Russell Ward. The correct affiliation 1 is: Research Centre for Public Health, Equity and Human Flourishing, Torrens University Australia, Adelaide, South Australia, Australia.
