# Supplementary material for: Understanding the quality of life of people living with HIV in rural and urban areas in Indonesia
Source: PLoS One. 2023 Jul 13;18(7):e0280087. doi: 10.1371/journal.pone.0280087 (PMC10343063; doi:10.1371/journal.pone.0280087)
Supplement: S1 File — (PDF) [file pone.0280087.s001.pdf]

Interview Guide

Environmental aspect

How would you describe your living condition or the environment where you live?

- Rural/urban?
- How do you feel about it? Satisfied/dissatisfied? Why? Please explain more.

What about your access to public transportation?

- Easy or not? / Available or not? Please explain.

What about your access to healthcare services?

- Any challenges/difficulties? Please explain more.
- What do you think about HIV care services? Please explain.

How would describe your financial condition following the HIV diagnosis?

- Do you think the infection influences you financially? How?
- Does financial condition influence your access to healthcare services? How/why? Please explain more.

Personal belief and psychological aspect

After your HIV diagnosis, what do you think about your future?

- Does the infection influence your thoughts and feelings about your future? How and why?
    - Have you ever experienced fear or worry about your future? Please elaborate more about things that make fear and worry about your future…
        - Rejection, the possibility of transmission to spouse

What is your thought about death?

- Is there any chance that the infection influences your thoughts and feelings about death? How and why? Please explain.
    - What do you think has changed your thoughts about death? Children, responsibility, long recovery period, etc….

Have you ever felt bothered by anybody who knows your HIV status? Why? Please elaborate….

Have you ever thought that your life is meaningful? Why? Please explain…

How would you describe your feelings/emotions in relation to the infection you have?

- Experiencing negative or positive emotions? How? Please explain more about them.
    - Stress, anxiety, depression, fear, etc….

- Feeling satisfied with yourself or not? Why? Please explain more about it.

Would mind describing things you have you done so far to enjoy your life?

How do you feel about your physical appearance? Please elaborate.

Social relationships

What do you think about your social relationships with others?

- Do you feel accepted or not? Why? Please explain more
- Do you feel satisfied with your personal relations or not? Why?

Have you ever experienced negative attitudes and behaviours from others after your HIV diagnosis? How and why? Please explain more.

- Within your family, the community where you live and healthcare settings.
- Do those attitudes and behaviours influence your social relationships? How and why?
    - Withdrawal from social relationships
    - Hiding HIV status
    - Etc…

How do you feel about the support from families and friends?

- Enough support or not? Why?

How would you describe your sex life after being diagnosed with HIV infection?

- Are there any changes you noticed? How and why? Please explain.
- Are you satisfied or not with your sex life after the diagnosis?
    - How does it influence your life and relationships? Why? Please explain more.

Level of independency and physical aspect

How would you describe your physical condition after the HIV diagnosis?

- Do you think the infection has affected you physically? How? Please explain more about it.
- How do you feel now physically? Please explain more

What other things do you think have contributed to the negative impacts of the infection on your physical health?

- Late diagnosis
- Late treatment, etc….

Does the infection prevent you from working or bother you physically? Please explain more about it.

- Reduction or working hours days?

- Inability to perform the work physically?

How do you maintain your daily physical activities i.e., study, work, and access to transportation, healthcare services, etc?

- Having enough energy to do them by yourself….
- Getting support from or being dependent on other people…..
